# Mesenteric panniculitis in a patient with rheumatoid arthritis

**DOI:** 10.1590/0100-3984.2017.0209

**Published:** 2019

**Authors:** Tiago Kojun Tibana, Rômulo Florêncio Tristão Santos, Denise Maria Rissato Camilo, Edson Marchiori, Thiago Franchi Nunes

**Affiliations:** 1 Universidade Federal de Mato Grosso do Sul (UFMS), Campo Grande, MS, Brazil.; 2 Universidade Federal do Rio de Janeiro (UFRJ), Rio de Janeiro, RJ, Brazil.

**Figure 1:**
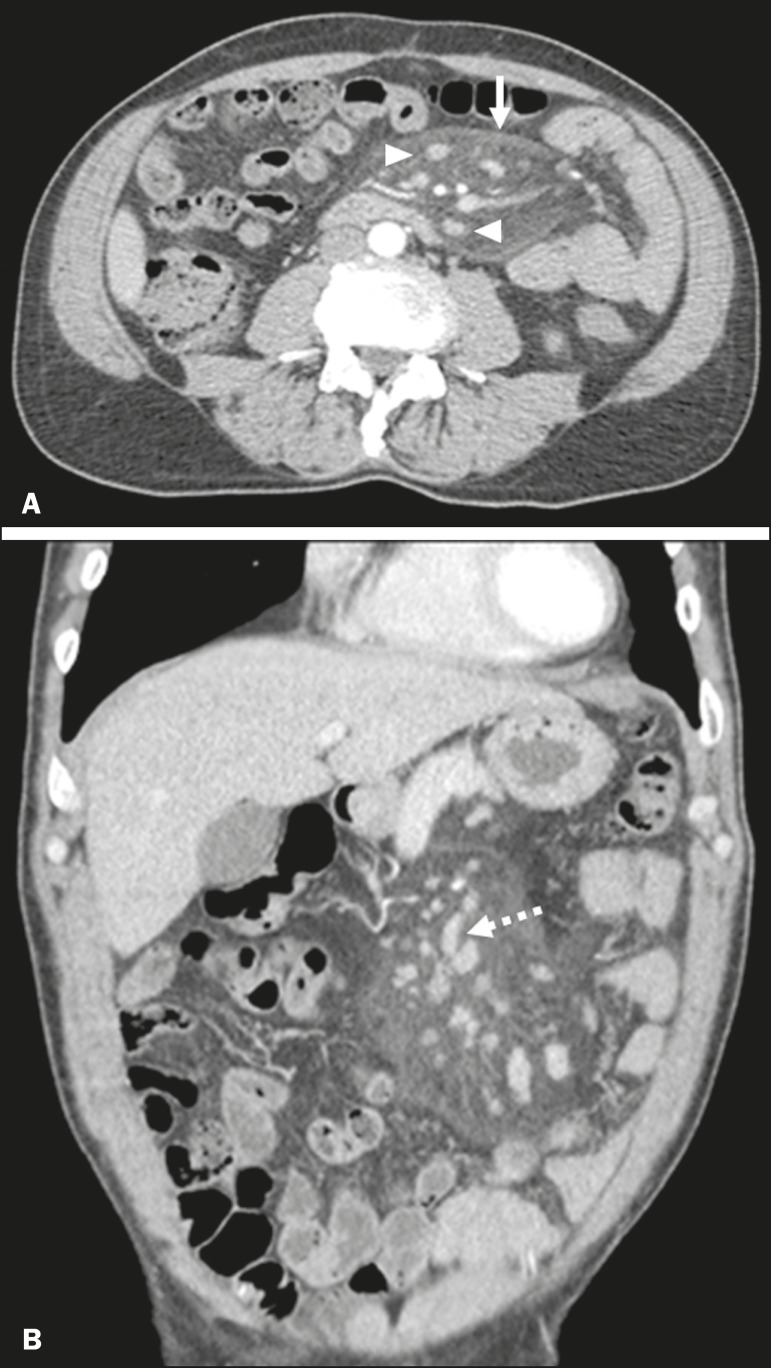
Axial and coronal CT (**A** and **B**, respectively) showing a heterogeneous expansile formation, with predominantly fat density, containing lymph nodes (arrowhead) and ectatic vascular structures (dotted arrow), partially delimited by a tumor pseudocapsule and extending from the root of the mesentery to the left iliac fossa.

Dear Editor,

A 63-year-old man presented with a four-month history of intermittent pain in the upper abdomen, progressively increasing in intensity, together with asthenia, nausea, and weight loss of 10 kg. He had been under treatment for rheumatoid arthritis (with methotrexate and prednisone) for seven years. Physical examination showed pain on deep palpation, together with a partially mobile, fibroelastic mass, in the left upper quadrant of the abdomen. Laboratory tests showed no significant changes, except for a slightly elevated erythrocyte sedimentation rate. Tumor markers were within the limits of normality. Computed tomography (CT) of the abdomen showed an expansile heterogeneous mass, with predominantly fat density, encompassing lymph nodes and containing ectatic vascular structures (Figure 1). Based on the clinical reports and the CT findings, the working diagnosis was mesenteric panniculitis. We chose to test our hypothesis by adjusting the dose of prednisone. The patient progressed satisfactorily, evolving to complete resolution of the symptoms.

Mesenteric panniculitis is a rare disease of as yet unknown etiology, characterized by chronic nonspecific inflammation involving the adipose tissue of the mesentery. It is most common in men between the fifth and sixth decades of life. It has been linked to a variety of conditions, such as infections, trauma, surgery, pancreatitis, mesenteric ischemia, and autoimmune disorders^(^^1^^-^^3^^)^. The symptoms of mesenteric panniculitis can be progressive, intermittent, or absent. Symptomatic patients can present with a palpable abdominal mass and nonspecific systemic manifestations, including abdominal pain, loss of appetite, asthenia, weight loss, and intestinal disorders of varying duration. Laboratory test results are nonspecific, including leukocytosis, anemia, and elevation of the erythrocyte sedimentation rate^(^^1^^,^^4^^,^^5^^)^.

In a variety of acute abdominal conditions, CT has been used as a diagnostic tool, as well as in the evaluation of treatment efficacy^(^^5^^-^^8^^)^. The CT findings depend on the stage of the disease and on whether the predominant component is inflammatory, fibrous, or adipose^(^^3^^)^. Mesenteric panniculitis usually presents as a heterogeneous mass with an adipose component, its density slightly increased by the local inflammatory process, together with linear bands of soft-tissue density (tumor pseudocapsule, detected in up to 50% of cases), as well as lymph node enlargement and mesenteric vascular ectasia^(^^9^^)^. Although the definitive diagnosis is established through laparoscopic biopsy^(^^5^^)^, that is not always necessary. Recent studies have shown that mesenteric panniculitis can be diagnosed on the basis of CT characteristics^(^^10^^,^^11^^)^.

There are as yet no treatments for mesenteric panniculitis that are considered totally efficacious^(^^1^^)^. The disease tends to resolve spontaneously. Pharmacological treatment is reserved for symptomatic cases and includes the use of corticosteroids, thalidomide, cyclophosphamide, progesterone, colchicine, and azathioprine. Surgical resection is limited to cases of intestinal obstruction and other complications, such as ischemia or high suspicion of malignancy^(^^1^^,^^11^^)^.
